# Supplementary material for: Canalized gene expression during development mediates caste differentiation in ants
Source: Nat Ecol Evol. 2022 Oct 3;6(11):1753–65. doi: 10.1038/s41559-022-01884-y (PMC9630140; doi:10.1038/s41559-022-01884-y)
Supplement: Supplementary file 1 — Supplementary Methods and references. [file 41559_2022_1884_MOESM1_ESM.pdf]

nature ecology & evolution

Article

https://doi.org/10.1038/s41559-022-01884-y

Canalized gene expression during development mediates caste differentiation in ants

In the format provided by the authors and unedited

Supplementary Method:

Table of Contents

Sample collection 1
Identifying developmental age and separating worker, gynes and males 2
Whole-body simultaneous RNA and DNA extraction for individual samples 3
Sample quality control 4
Transcriptome profiling and normalization 4
Correction for batch effects 5
Testing the influence of technical variation on individual transcriptomic variation 6
Validating the accuracy of BPA 6
Testing the influence of sex on early developmental transcriptomes in *M. pharaonis* 7
Functional enrichment and loss of function enrichment analyses. 7
Detection of orthologs and homologs across species 7

Sample collection

M. pharaonis

Two colonies (D03 and 4030) of *M. pharaonis* were used in this study. Both were derived from interbreeding a global variety of genetic lineages and have been kept in captivity at the University of Copenhagen since 2004 [1], so they had substantial genetic variation in spite of having been propagated as pure lines since collection. Colonies were kept at 27 °C and 50 % relative humidity throughout the experiment. Individual samples were collected between January 2017 and March 2018. Because production of sexuals only occurs in colonies without queens (Q-) [2], Q- sub-colonies were set up, from which all larvae, pupae and adults were collected. Collections of 1st-instar larvae were made more than 8 days after queens had been removed to ensure these larvae would include sexual-destined individuals following the insights obtained earlier [2,3].

To collect samples of developing embryos, sub-colonies were set up by isolating approximate 10 queens and 100 workers. Queens were left to lay egg for 12 hours (an average queen produces ca. 1-2 eggs per hour) [4], after which the queens were removed. At least 20 eggs were then collected for each of the nine time points throughout embryo development: 0-12, 12-24, 36-48, 60-72, 84-96, 108-120, 132-144, 156-168 and 180-192 hours after oviposition (egg laying). At 192 hours after oviposition, embryos are in the last stages of embryo development (stage 14 to 17), so we were sure to have covered the entire egg stage of embryonic development. To minimize disturbance, a series of sub-colonies for embryo collection were setup independently for each developmental time point, so each sub-colony was only used once.

Individuals sampled as egg, larva, pupa or callow (adult/imago just after eclosion) were carefully collected, photographed (except eggs) (Leica MZ125 microscope) and stored individually, before being flash frozen in liquid nitrogen and stored at -80 °C until extraction.

A. echinatior

Three colonies of *A. echinatior* were used for this experiment, all collected in Gamboa, Panama (Ae150 collected on 19-04-2001, Ae394 collected on 28-04-2009 and Ae506 collected on 10-05-2011). Colonies were kept at 25 °C and 70 % relative humidity and were fed bramble leaves, rice and apples tree time a week. Individual samples were collected between March 2016 and November 2018. Larvae, pupae and callows were carefully removed from the fungus garden and individually photographed (Canon EOS 7D MarkII with macro lens EF 100mm). Samples were stored as in *M. pharaonis*.

D. melanogaster

The inbred wild-type genetic background *Canton-S* of *Drosophila melanogaster* were used for the experiment. Fly cultures were kept at 25 °C and 60% relative humidity throughout the experiment, with a 12-hour/12-hour light/dark cycle on standard *Drosophila* medium (commercial NutriFly medium, “Bloomington” recipe; Gennessee Scientific). Individual samples were collected between July 2017 and April 2018. Prior to egg collections the adult flies were purged of retained eggs by incubating in egg-laying chambers overnight with apple-juice/agar dishes covered with yeast paste. Eggs for experimental use were collected by swapping with a new freshly yeasted dish and incubating for 2 hours. Embryos were left on these plates for 24 hours until hatching, after which larvae were manually transferred with a fine probe to culture tubes of standard medium. Flies of the selected life stages were individually removed from culture media, rinsed in deionized water. Samples were stored as in *M. pharaonis*.

Identifying developmental age and separating worker, gynes and males

For both ant species, individual samples of larvae and pupae were laterally photographed during collection, after which body length and head capsule width (only for larvae) were measured using Adobe Photoshop CC 19.1.6 or ImageJ 1.53c. To distinguish males and females, DNA from each individual sample were extracted during the RNA extraction process (see *RNA and DNA extraction for individual samples*). Developmental age, castes and sexes of samples were identified with the following procedures:

M. pharaonis

Developmental stages of *M. pharaonis* were estimated based on their body length and morphological characters (see Extended Data Table 2) [2,5]. Developmental stages of early and late pupae were determined by visual assessment of cuticle colour (males turn black and females turn brown), using a colour scale obtained from age-controlled pupae. Because pupal deployment takes 12 days at 27 °C, early pupae and late pupae were collected between 0-2 days and between 10-12 days after the onset of pupation, respectively. Callow adult ants were collected based on their light cuticle colour, corresponding to ages a few days after eclosion.

From the 2nd-instar larval onwards, workers and sexuals (males and gynes) can be distinguished by morphological traits (see Extended Data Table 2). To further distinguish males and gynes (females) among the 2nd and 3rd-instar larval and pre-pupal sexuals, microsatellite genotyping (on the extracted DNA) was employed to identify whether an individual was a haploid (male) or diploid (female), using five highly polymorphic nuclear microsatellite loci, with primer set: Mp4, Mp8, Mph2, Mph23 and Mph9 [1]. Repeat lengths of microsatellite loci were determined on an Applied Biosystems, Hitachi 3130xl, Genetic Analyzer and analysed using GeneMapper 4.0 software (Applied Biosystems).

A. echinatior

Developmental stages of larvae were estimated based on their body length and morphological characters (see Extended Data Table 2) [6], also see: https://megalomyrmex.osu.edu/temp/acro-larva-key/). The ages of pupae and callow adults were estimated by visual assessment of cuticle colour. Pupal development lasts between 20 – 27 days (own lab observations; see also [7]), so our samples of early and late pupae refer to the beginning and end of this range. Castes and sexes of 3rd and 4th-instar larvae and pre-pupae were distinguished based on the hair morphology (see Extended Data Table 2). To further distinguish males and females among 1st and 2nd-instar larvae where morphological markers for caste identity are not available, microsatellite genotyping were used to identify whether an individual was haploid (male) of diploid (female), using four highly polymorphic nuclear microsatellite loci (primer set: Acrin02, Acrin05, Acrin22, Acrin29 [6]. Repeat lengths of microsatellite loci were determined as in *M. pharaonis*.

D. melanogaster

For *D. melanogaster*, chronological age was used to collect selected developmental stages (Extended Data Table 1). Genotyping was used to identify the sex of 2nd and 3rd-instar larvae and pupae. The sex-chromosome male fertility gene *kl-5* (primer Kl5-F8 and Kl5-R6) was selected as identifier for the males and used the autosomal *tpi* gene as a positive control (primer Tpi-F and Tpi-R) [8]. PCR amplifications were performed on the extracted DNA and a presence/absence gel-electrophoresis analysis was used to identify the sex of larvae.

Whole-body simultaneous RNA and DNA extraction for individual samples

M. pharaonis

For embryos and 1st-instar larvae, RNAs were extracted using PicoPure RNA Isolation Kits (Thermo Fisher), following the standard protocol, except embryos were first squashed with the blunt tip of a pulled glass capillary in order to homogenize and release the cell contents. For 2nd and 3rd-instar larvae, pre-pupae, pupae and callows, RNAs were extracted with RNeasy Plus Micro kits (Qiagen). During the lysis process, 5 mm stainless steel beads were used and Reagent DX (Qiagen) and 2-Mercaptoethanol were added to the lysis buffer. DNAs were retrieved from 2nd and 3rd-instar larvae and pre-pupae by eluting the gDNA eliminator spin columns from the RNeasy Plus Micro kit. DNAs were recovered from the gDNA

elimination column in the RNeasy Plus Micro kit by washing the column with AW1 and AW2 and eluting with AE buffer.

A. echinatior

RNAs and DNAs of 1st and 2nd-instar larvae in *A. echinatior* were extracted with RNeasy Plus Micro kits (Qiagen), following the same procedure as in *M. pharaonis*. For later stages, samples were sent to BGI sequencing service (Hong Kong) for whole body RNA extraction. Briefly, individuals were homogenized using a liquid nitrogen chilled mortar and pestle immediately following removal from the freezer. RNAs were extracted using TRIzol Reagent (Invitrogen) following a standard RNA-isolation protocol. For large individuals, a fraction of the homogenate was used so as not to exceed ~10% of TRI Reagent volume [9].

D. melanogaster

RNA of embryos and 1st-instar larvae were extracted with the PicoPure RNA Isolation Kit (ThermoFisher). RNA and DNA from all other stages were extracted using the RNeasy Plus Micro kit (Qiagen) following the same procedure as in *M. pharaonis*.

Sample quality control

Besides removing samples with low RNA quality (RIN < 4) before sequencing, samples with poor RNAseq quality were also discarded after sequencing, either due to technical biases, e.g. cDNA library construction or sequencing, or when being biological outliers, e.g. delayed growth or dead samples. Within each species, the transcriptome (normalized gene expression profile) similarities between any two samples were calculated by measuring their pairwise Spearman's correlation coefficients. In the comparisons that followed for each developmental stage, samples were then considered to be outliers if their within-stage transcriptome similarity was low (Spearman's correlation coefficient < 0.8) [10]. Following these criteria, 12 (out of 879) samples were discarded for *M. pharaonis*, including six embryonic samples, one 2nd instar, one 3rd instar and two adult workers, one 3rd instar gyne, and one 3rd instar male. For *A. echinatior*, 9 (out of 599) samples were discarded, including one 2nd instar larva (of unknown caste), one early and two late-stage pupal workers, and one 3rd instar, three 4th instar and one prepupal gynes. For *D. melanogaster*, 12 (out of 493) samples were discarded, including 11 embryonic and one 1st instar larval samples.

Transcriptome profiling and normalization

RNAseq data were first pre-processed with SOAPnuke (*version* 2.0.7) to remove adapters and to filter low quality reads [11]. Filtered RNAseq data were then mapped onto the transcriptome index of the corresponding species with *Salmon* (mapping-based mode) (*version* 1.4.0) [12], producing raw transcriptome abundance profiles (numbers of mapped reads for all annotated genes) for each of the individual samples.

The following genome assemblies and transcriptomic indexes were used for transcriptomic quantifications: *Monomorium pharaonis* (Chromosome level assembly GCF_013373865.1; NCBI annotation release 102), *Acromyrmex echinatior* (In-house assembly provided by

Global Ant Genomics Alliance [13]; In-house annotation with GeMoMa (*version* 1.7.1) [14], using *Monomorium pharaonis*, *A. echinatior* [NCBI annotation release 100] and *Solenopsis invicta* [NCBI annotation release 103] as transcriptomic annotation reference), and *Drosophila melanogaster* (Genome assembly GCF_000001215.4; Flybase annotation release 6.32).

The raw transcriptome abundances (read counts or Transcripts Per Kilobase Million (TPM), used for cases of plotting gene expression levels) were log 2 transformed (after adding 1 to each score to avoid log 0) and then quantile normalized across samples to ensure that the overall distributions of gene expression levels remained the same across samples, which minimized the effects of any technical artefacts that might have occurred [15].

To ensure that results were robust to different normalization methods, variance stabilizing transformation (VST) [16] was also applied for each method before comparison. This procedure included three steps:(1) estimating the size factor (sequencing depth) of the raw transcriptome abundances for each of the samples; (2) estimating the dispersion-to-mean relationships across transcriptomes obtained from the raw transcriptome abundances after scaling by the size factor for each sample; and (3) normalising the transcriptome abundances for all samples using the fitted dispersion-to-mean relationships across transcriptomes. The resulting VST normalization thus takes both sequencing depth and dispersion-to-mean relations into account, so the normalized transcriptome abundances become comparable across all samples and all genes with different expression levels [16].

We observed that these two normalization methods gave similar results, so quantile normalization was mainly used for the analyses except for the quantifications of within-stage transcriptome variation and between-caste variation, where VST was used to obtain a robust set of measurements.

Correction for batch effects

Although RNA extraction, cDNA library construction and sequencing were all done with similar procedures (except for embryos and 1st instar samples for which a different extraction kit was used), we noticed a systematic expression difference for samples that had been sequenced before July 2018 (Batch A; 925 samples), before April 2019 (Batch B; 329 samples) and afterwards (Batch C;169 samples), producing three technical batches that could potentially confound the comparative analyses, especially among samples in *A. echinatior*. However, we found that removing batch B and C did not significantly impact our conclusions and therefore retained all samples to attain a maximally complete coverage of all developmental stages.

To correct for the batch effects, we used the body length measurements and the even distribution of batches across developmental stages. Batch effects on the gene expression level were estimated with the following linear regression model:

$$Exp_{gene} \sim batch_{gene} + age * \left(caste + log(bodylength)\right)$$

We assumed that the batch effects on each gene's expression level were consistent between castes and across developmental stages and that the effect of body length on gene expression level was consistent between castes within the same developmental stage. A linear regression model was then used to estimate putative batch effects. After partialing out the variance component that could have been due to batch effect, we obtained gene expression levels of same-caste and same-stage individuals that were similar across batches, and an expected overall transcriptomic expression pattern where individuals of the same caste and same developmental stage clustered together regardless of batch. These adjusted gene expression levels were then used for all downstream analyses, except for transcriptome variation analyses. Differences in expression variation cannot be corrected by regression-based batch corrections, so we excluded samples of the minority batch for these analyses.

Testing the influence of technical variation on individual transcriptomic variation
Technical variation can be a major confounding factor for individual transcriptomic variation detection. We found that RNA yields in larvae are both lower and more variable than in pupae. This is not surprising because (1) RNA yields are expected to be correlated with the body mass of samples, and (2) larvae are still actively growing and thus have higher body mass variation than pupae. However, RNA yield variation cannot technically explain our observed transcriptomic data. First, RNA-seq is a proportional measurement that quantifies relative gene expression levels, which should be independent of RNA yield during extraction. Second, although we found an association (Pearson correlation coefficient = 0.53; $P = 0.11$ in a two-sided test), the RNA yield variation did not match the variation in developmental potential in our data. For example, while there was a higher RNA yield variation in 2nd instar gynes than in 2nd instar workers, their developmental potential variation exhibited an opposite pattern. Third, all else being equal, compared to RNA yield, RNA integrity number (RIN), a widely used statistic that assesses in vitro RNA degradation, is a better quantification of technical variation at RNA level, and we found no correlation between RIN variation and observed developmental potential variation (Pearson correlation coefficient = -0.02; $P = 0.95$ for two-sided test).

We have also taken care of batch effects. 498 samples that were generated in different sequencing batches (identified by PCA of transcriptomes and further confirmed by examining the experimental metadata) were retained for differentially expressed gene analysis (after batch correction), but excluded from transcriptomic variation analyses to minimize the effect of batch variation. Therefore, we are confident that the observed pattern of transcriptomic variation that we report represent biological variation among samples with minimal effects of technical artifacts.

Validating the accuracy of BPA
BPA was tested on data sets of five later developmental stages for which caste identity was known and found that accuracies of single stage prediction were 86-100% (Extended Data Fig. 2c). *M. pharaonis* individuals beyond the pre-pupal stage could all be correctly assigned to their observed caste identities, but we observed a gradual decline in BPA accuracy when we conducted backward caste reconstructions for earlier stages. For example, BPA-accuracy

dropped to 73% when working backwards from 2nd instar larvae. This is as expected because prediction errors increase multiplicatively across sequential rounds and because transcriptomic differences between castes are likely to be less pronounced in earlier developmental stages. We therefore only used BPA predictions in larval stages where experimental validation was possible.

Testing the influence of sex on early developmental transcriptomes in *M. pharaonis*

The small biomass of 1st instar larvae (and of the earlier embryos) precluded extracting DNA for microsatellite genotyping to remove haploid male individuals. However, we found that validated 2nd instar larval transcriptomes for gynes and males were very similar when compared to significantly different 2nd instar worker larvae (Extended Data Fig. 2f), indicating that early transcriptomic differentiation primarily reflects differences between reproductive (gynes and males) and sterile (worker) developmental trajectories.

Functional enrichment and loss of function enrichment analyses.

Functional enrichment and loss of function enrichment analyses for target gene sets in ants were done based on their homologs in *D. melanogaster*. Protein sequences of *M. pharaonis* and *A. echinatior* were first annotated with their *Drosophila* homologs' Gene Ontology (GO), Kyoto Encyclopedia of Genes and Genomes (KEGG) information and allele loss of function phenotypes from Flybase [17]. Functional enrichments were then obtained by over-representation tests with the clusterProfiler package (*version* 4.0.5) [18], evaluating the percentage of genes in particular functional categories (e.g. biological functions or pathways) among the target gene set, e.g. caste DEGs. Functional categories were accepted as significantly over-represented (enriched) if their P values < 0.05 and q values < 0.1.

The enriched functional terms contained redundant terms with similar gene members and functions. To provide a concise description of gene functions, the enriched terms were further clustered based on their gene membership similarities, calculated by the ratio of the number of shared gene members and the number of total non-redundant gene members. These similarities range between 0 to 1, with 1 indicating a complete overlap of gene members. For each term cluster, the most significant term, ranked by their P values in the enrichment analysis, was then used as the representative term. A full list of enriched functional terms is provided in Supplementary table 5.

Detection of orthologs and homologs across species

Orthologs among *M. pharaonis*, *A. echinatior* and *D. melanogaster* were identified with Orthofinder (*version* 2.5.4), a phylogeny-based ortholog inference method [19]. To infer orthologs, the proteomes of 17 species (16 from NCBI and one, *A. echinatior,* from our in-house annotated proteome as its PacBio genome annotation is not yet available in NCBI) were used. These included 11 species of Hymenoptera, including four ant species (*A. echinatior*, *M. pharaonis*, *Ooceraea biroi* and *Harpegnathos saltator*), three bee species (*Apis mellifera*, *Bombus terrestris* and *Osmia lignaria*), three wasps species (*Polistes dominula*, *Vespa mandarinia* and *Nasonia vitripennis*) and the sawfly *Cephus cinctus*. Six non-hymenopteran species, i.e. two fly species (*D. melanogaster* and *D. willistoni*), one

mosquito (*Aedes albopictus*), one moth (*Bombyx mori*), one beetle (*Photinus pyralis*) and one butterfly (*Maniola hyperantus*) were also included. For genes with multiple isoforms, the longest proteins were always used as the representative sequence.

The default parameters in OrthoFinder were then used to detect orthologs, except when using BLAST (ver. 2.12.0) [20] for similar sequence searching and multiple sequence alignment for gene tree inference, which provided the best ortholog inference performance according to the benchmark test [19]. For genes with multiple orthologs, the most similar ortholog was used but information for all orthologs is provided in the Supplementary materials. Orthologs were used for downstream comparative transcriptomic analyses, functional enrichment analyses, and tissue-specific relative expression abundance estimation.

Supplementary References:

1. Schmidt, A. M., D'Ettorre, P. & Pedersen, J. S. Low levels of nestmate discrimination despite high genetic differentiation in the invasive pharaoh ant. *Front Zool* 7, (2010).

2. Edwards, J. P. Caste regulation in the pharaoh's ant Monomorium pharaonis: recognition and cannibalism of sexual brood by workers. *Physiol Entomol* 16, 263–271 (1991).

3. Boonen, S. & Billen, J. Caste regulation in the ant Monomorium pharaonis (L.) with emphasis on the role of queens. *Insect Soc* 64, 113–121 (2017).

4. Peacock, A. D., Haul, D. W., Smith, I. C. & Goodfellow, A. The Biology and Control of the Ant Fest Monomorium pharaonis (L.). *The Biology and Control of the Ant Fest Monomorium pharaonis (L.).* (1950).

5. Pontieri, L. *et al.* From egg to adult: a developmental table of the ant Monomorium pharaonis. *Biorxiv* 2020.12.22.423970 (2020) doi:10.1101/2020.12.22.423970.

6. Adams, R. M. M. *et al.* Hairs distinguish castes and sexes: Identifying the early ontogenetic building blocks of a fungus-farming superorganism (Hymenoptera: Formicidae). *Myrmecological News.* 31, 201–216 (2021).

7. Dijkstra, M. B. & Boomsma, J. J. The economy of worker reproduction in Acromyrmex leafcutter ants. *Anim Behav* 74, 519–529 (2007).

8. Dyer, K. A., White, B. E., Bray, M. J., Piqué, D. G. & Betancourt, A. J. Molecular Evolution of a Y Chromosome to Autosome Gene Duplication in Drosophila. *Mol Biol Evol* 28, 1293–1306 (2011).

9. Hunt, B. G. & Goodisman, M. A. D. Evolutionary variation in gene expression is associated with dimorphism in eusocial vespid wasps. *Insect Mol Biol* 19, 641–652 (2010).

10. Encode-Consortium. ENCODE Guidelines and Best Practices for RNA-Seq. Preprint at https://www.encodeproject.org/documents/cede0cbe-d324-4ce7-ace4-

f0c3eddf5972/@@download/attachment/ENCODE%20Best%20Practices%20for%20RNA_v2.pdf (2016).

11. Chen, Y. *et al.* SOAPnuke: a MapReduce acceleration-supported software for integrated quality control and preprocessing of high-throughput sequencing data. *Gigascience* 7, 1--6 (2018).

12. Patro, R., Duggal, G., Love, M. I., Irizarry, R. A. & Kingsford, C. Salmon provides fast and bias-aware quantification of transcript expression. *Nat Methods* 14, 417--419 (2017).

13. Boomsma, J. J. *et al.* The Global Ant Genomics Alliance (GAGA). *Myrmecological News* 25, 61–66 (2017).

14. Keilwagen, J. *et al.* Using intron position conservation for homology-based gene prediction. *Nucleic Acids Research* 44, e89–e89 (2016).

15. Zyprych-Walczak, J. *et al.* The Impact of Normalization Methods on RNA-Seq Data Analysis. *Biomed Res Int* 2015, (2015).

16. Love, M. I., Huber, W. & Anders, S. Moderated estimation of fold change and dispersion for RNA-seq data with DESeq2. *Genome Biol* 15, (2014).

17. Larkin, A. *et al.* FlyBase: updates to the Drosophila melanogaster knowledge base. *Nucleic Acids Res* 49, D899–D907 (2020).

18. Wu, T. *et al.* clusterProfiler 4.0: A universal enrichment tool for interpreting omics data. *Innovation* 2, 100141 (2021).

19. Emms, D. M. & Kelly, S. OrthoFinder: phylogenetic orthology inference for comparative genomics. *Genome Biol* 20, 238 (2019).

20. Altschul, S. F., Gish, W., Miller, W., Myers, E. W. & Lipman, D. J. Basic local alignment search tool. *J Mol Biol* 215, 403–410 (1990).
