# Supplementary material for: Canalized gene expression during development mediates caste differentiation in ants
Source: Nat Ecol Evol. 2022 Oct 3;6(11):1753–65. doi: 10.1038/s41559-022-01884-y (PMC9630140; doi:10.1038/s41559-022-01884-y)
Supplement: Supplementary file 2 — Reporting Summary [file 41559_2022_1884_MOESM2_ESM.pdf]

nature portfolio

Corresponding author(s): Bitao Qiu
Jacobus Jan Boomsma
Guojie Zhang

Last updated by author(s): Jul 20, 2022

Reporting Summary

Nature Portfolio wishes to improve the reproducibility of the work that we publish. This form provides structure for consistency and transparency in reporting. For further information on Nature Portfolio policies, see our Editorial Policies and the Editorial Policy Checklist.

Statistics

For all statistical analyses, confirm that the following items are present in the figure legend, table legend, main text, or Methods section.

n/a	Confirmed	
☐	☒	The exact sample size (*n*) for each experimental group/condition, given as a discrete number and unit of measurement
☐	☒	A statement on whether measurements were taken from distinct samples or whether the same sample was measured repeatedly
☐	☒	The statistical test(s) used AND whether they are one- or two-sided<br>*Only common tests should be described solely by name; describe more complex techniques in the Methods section.*
☐	☒	A description of all covariates tested
☐	☒	A description of any assumptions or corrections, such as tests of normality and adjustment for multiple comparisons
☐	☒	A full description of the statistical parameters including central tendency (e.g. means) or other basic estimates (e.g. regression coefficient) AND variation (e.g. standard deviation) or associated estimates of uncertainty (e.g. confidence intervals)
☐	☒	For null hypothesis testing, the test statistic (e.g. *F*, *t*, *r*) with confidence intervals, effect sizes, degrees of freedom and *P* value noted<br>*Give P values as exact values whenever suitable.*
☒	☐	For Bayesian analysis, information on the choice of priors and Markov chain Monte Carlo settings
☒	☐	For hierarchical and complex designs, identification of the appropriate level for tests and full reporting of outcomes
☒	☐	Estimates of effect sizes (e.g. Cohen's *d*, Pearson's *r*), indicating how they were calculated

Our web collection on statistics for biologists contains articles on many of the points above.

Software and code

Policy information about availability of computer code

Data collection

No software was used to collect data.

Data analysis

Custom code has been deposited in: https://github.com/BitaoQiu/devo-ants

The following software were used to analysis the data:

Morphological measurements: Adobe Photoshop CC 19.1.6 & ImageJ 1.53c
Imaging processing for fluorescence in situ hybridization: Fiji/ImageJ 1.53c
Microsatellite loci analysis: GeneMapper 4.0

Genome analyses:
Genome annotation: GeMoMa (ver. 1.7.1)
Ortholog detection: Orthofinder (ver. 2.5.4)
Sequence alignment: BLAST (ver. 2.12.0)
Identification of phylogenetic origin of genes:
Multiple sequence alignment: T-coffee (ver. 13.45.0)
Gene tree construction: IQ-TREE (ver. 2.1.4)

RNAseq analyses:
RNAseq reads quality control: SOAPnuke (ver. 2.0.7)
Transcriptome profiling: Salmon (ver. 1.4.0)
RNAseq read normalization, Variance stabilizing transformation, and DEG detection: DESeq2 (ver. 1.32.0)
Construction of developmental trajectory network from transcriptomes: igraph (ver. 1.2.9)

Between-stage expression level normalization: sva (ComBat) (ver. 3.40.0)
Threshold regression model: chngpt (ver. 2021.5-12)
Robust linear regression: rlm from MASS (ver. 7.3)
Functional enrichment analysis: clusterProfiler (ver. 4.0.5)

For manuscripts utilizing custom algorithms or software that are central to the research but not yet described in published literature, software must be made available to editors and reviewers. We strongly encourage code deposition in a community repository (e.g. GitHub). See the Nature Portfolio guidelines for submitting code & software for further information.

Data

Policy information about availability of data

All manuscripts must include a data availability statement. This statement should provide the following information, where applicable:

- Accession codes, unique identifiers, or web links for publicly available datasets
- A description of any restrictions on data availability
- For clinical datasets or third party data, please ensure that the statement adheres to our policy

RNAseq data that support the findings of this study have been deposited in GenBank with the BioProject accession codes PRJNA767561 (https://dataview.ncbi.nlm.nih.gov/object/PRJNA767561?reviewer=knbfs1f376d1idqf5crfn1ke5s)

Field-specific reporting

Please select the one below that is the best fit for your research. If you are not sure, read the appropriate sections before making your selection.

☒ Life sciences ☐ Behavioural & social sciences ☐ Ecological, evolutionary & environmental sciences

For a reference copy of the document with all sections, see nature.com/documents/nr-reporting-summary-flat.pdf

Life sciences study design

All studies must disclose on these points even when the disclosure is negative.

Sample size

In total 1921 individual transcriptome samples, including: 819, 629, and 491 samples from M. pharaonis, A. echinatior and D. melanogaster, respectively, which secured having ca. 30 samples per stage per caste and a minimum of 30 samples per stage when caste phenotypes could not be determined.

Data exclusions

63 samples were removed due to poor RNAseq quality (having within-stage Spearman correlation coefficients with other transcriptomes < 0.8).

Although RNA extraction, cDNA library construction and sequencing were all done with similar procedures (except for embryos and 1st instar samples for which we used a different extraction kit), we noticed a systematic expression difference for samples that had been sequenced before July 2018 (Batch A; 925 samples), before April 2019 (Batch B; 329 samples) and afterwards (Batch C;169 samples), producing three technical batches that could potentially confound the comparative analyses, especially among the A. echinatior samples.

We therefore excluded Batch B and C from cross-stage variation comparison while retained them for between-caste expression difference analysis, because the latter analysis can be adjusted for batch-effects by partial linear regression (see Methods for details).

Replication

We successfully verified our computational predictions of early caste marker genes with RNA fluorescence in situ hybridization.

We used quantitative reverse transcription PCR (RT-qPCR) to verify the top 10 canalized genes (genes with increasing between-caste expression divergence) and achieved matches in all cases.

For the roles of JH and Freja in caste canalization regulation, a minimum of four experimental replications have been conducted and all produced consistent results that confirmed our transcriptomic findings.

Randomization

Samples of each ant species were randomly collected from the same two (M. pharaonis, D03 and 4030) or three (A. echinatior, Ae150, Ae394 and Ae506) colonies.

Samples of the same developmental stages were randomized and processed with the same experimental procedures (RNA extraction, cDNA library construction and RNA sequencing), so that gyne and worker samples of the same stage were always randomized with minimal technical batch effect.

For the RNAi, JHA and precocene I experiments, experimental and control group individuals were randomly collected from the same colonies. During experiments, experimental and control group individuals were fed with the same food but reared separately, because we needed to add workers to take care of the larvae, which would have become mixed (between experimental and control groups) if they had been reared together.

Blinding

Blinding was not relevant to our study because we were examining the gyne-worker caste differentiation process, where caste identities need to be identified beforehand. For BPA (computational prediction of caste fate) and experimental validation of caste marker genes among early stage individuals, individuals' caste fates were blind to the experimenters.

Reporting for specific materials, systems and methods

We require information from authors about some types of materials, experimental systems and methods used in many studies. Here, indicate whether each material, system or method listed is relevant to your study. If you are not sure if a list item applies to your research, read the appropriate section before selecting a response.

Materials & experimental systems

n/a	Involved in the study
☒	☐ Antibodies
☒	☐ Eukaryotic cell lines
☒	☐ Palaeontology and archaeology
☐	☒ Animals and other organisms
☒	☐ Human research participants
☒	☐ Clinical data
☒	☐ Dual use research of concern

Methods

n/a	Involved in the study
☒	☐ ChIP-seq
☒	☐ Flow cytometry
☒	☐ MRI-based neuroimaging

Animals and other organisms

Policy information about studies involving animals; ARRIVE guidelines recommended for reporting animal research

Laboratory animals

We used Monomorium pharaonis from two colonies (D03 and 4030), both derived from interbreeding a global variety of genetic lineages in 2004, after which colonies have been kept in captivity at the University of Copenhagen at 27 °C and 50 % relative humidity throughout the experiments. Individual samples were collected between January 2017 and March 2018. Sample collection included all developmental stages (from 0-3 hour embryos to newly emerged adults) and gynes, workers and males of the experimental colonies. Caste identities of individuals were identified with morphological characters (for larvae, pupae, and adults), and sexes of individuals were identified with morphological characters (for pupae and adults) or microsatellite genotyping (for larvae) to determine ploidy.

We used Acromyrmex echinatior from three colonies (Ae150, Ae394 and Ae506), all collected in Gamboa, Panama between 2001 to 2011. Colonies were kept at 25 °C and 70 % relative humidity and were fed bramble leaves, rice and apples tree time a week. Individual samples were collected between March 2016 and November 2018. Sample collection included major developmental stages (from 1st instar larvae to newly emerged adults) and gynes, small/medium/large workers and males. Caste identities of individuals were identified with morphological characters and sexes of individuals were identified with morphological characters (for late stage larvae, pupae and adults) or microsatellite genotyping (for 1st and 2nd instar larvae).

We used Drosophila melanogaster with inbred wild-type genetic background Canton-S. Fly cultures were kept at 25 °C and 60% relative humidity throughout the experiment, with a 12-hour/12-hour light/dark cycle on standard Drosophila medium. Sample collection included all developmental stages (from 1 hour embryos to newly emerged adults) and both sexes of the experimental animals. Sexes of individuals were identified with morphological characters (for adults) or genotyping (for larvae and pupae).

Wild animals

Provide details on animals observed in or captured in the field; report species, sex and age where possible. Describe how animals were caught and transported and what happened to captive animals after the study (if killed, explain why and describe method; if released, say where and when) OR state that the study did not involve wild animals.

Field-collected samples

For laboratory work with field-collected samples, describe all relevant parameters such as housing, maintenance, temperature, photoperiod and end-of-experiment protocol OR state that the study did not involve samples collected from the field.

Ethics oversight

Identify the organization(s) that approved or provided guidance on the study protocol, OR state that no ethical approval or guidance was required and explain why not.

Note that full information on the approval of the study protocol must also be provided in the manuscript.
